# Priming of Hypothalamic Ghrelin Signaling and Microglia Activation Exacerbate Feeding in Rats’ Offspring Following Maternal Overnutrition

**DOI:** 10.3390/nu11061241

**Published:** 2019-05-31

**Authors:** Roger Maldonado-Ruiz, Marcela Cárdenas-Tueme, Larisa Montalvo-Martínez, Roman Vidaltamayo, Lourdes Garza-Ocañas, Diana Reséndez-Perez, Alberto Camacho

**Affiliations:** 1 Department of Biochemistry, College of Medicine, Universidad Autónoma de Nuevo León, Monterrey, C.P. 64460, México; rogalmalruiz@gmail.com (R.M.-R.); lj_montalvo.mtz@hotmail.com (L.M.-M.); 2 Neurometabolism Unit. Center for Research and Development in Health Sciences, Universidad Autónoma de Nuevo León, Monterrey, C.P. 64460, México; 3 Department of Cell Biology and Genetics, College of Biological Sciences, Universidad Autónoma de Nuevo León, Monterrey, C.P. 64460, México; marcela.cdns@gmail.com (M.C.-T.); diaresendez@gmail.com (D.R.-P.); 4 Department of Basic Science, School of Health Sciences, Universidad de Monterrey, San Pedro Garza, N.L. 66238, México; roman.vidaltamayo@udem.edu; 5 Departamento de Farmacologia y Toxicología, College of Medicine, Universidad Autonoma de Nuevo Leon, Monterrey, C.P. 64460, México; logarza@live.com.mx

**Keywords:** ghrelin, hypothalamic inflammation, microglia, nutritional programing

## Abstract

Maternal overnutrition during pregnancy leads to metabolic alterations, including obesity, hyperphagia, and inflammation in the offspring. Nutritional priming of central inflammation and its role in ghrelin sensitivity during fed and fasted states have not been analyzed. The current study aims to identify the effect of maternal programming on microglia activation and ghrelin-induced activation of hypothalamic neurons leading to food intake response. We employed a nutritional programming model exposing female Wistar rats to a cafeteria diet (CAF) from pre-pregnancy to weaning. Food intake in male offspring was determined daily after fasting and subcutaneous injection of ghrelin. Hypothalamic ghrelin sensitivity and microglia activation was evaluated using immunodetection for Iba-1 and c-Fos markers, and Western blot for TBK1 signaling. Release of TNF-alpha, IL-6, and IL-1β after stimulation with palmitic, oleic, linoleic acid, or C6 ceramide in primary microglia culture were quantified using ELISA. We found that programmed offspring by CAF diet exhibits overfeeding after fasting and peripheral ghrelin administration, which correlates with an increase in the hypothalamic Iba-1 microglia marker and c-Fos cell activation. Additionally, in contrast to oleic, linoleic, or C6 ceramide stimulation in primary microglia culture, stimulation with palmitic acid for 24 h promotes TNF-alpha, IL-6, and IL-1β release and TBK1 activation. Notably, intracerebroventricular (i.c.v.) palmitic acid or LPS inoculation for five days promotes daily increase in food intake and food consumption after ghrelin administration. Finally, we found that i.c.v. palmitic acid substantially activates hypothalamic Iba-1 microglia marker and c-Fos. Together, our results suggest that maternal nutritional programing primes ghrelin sensitivity and microglia activation, which potentially might mirror hypothalamic administration of the saturated palmitic acid.

## 1. Introduction

Maternal obesity or maternal overnutrition during pregnancy and lactation programs an adverse uterine milieu leading to defects in organ function and metabolism in offspring [1,2]. Programing involves a new setting of peripheral and central pathways including energy expenditure and appetite regulation, which potentially increase the susceptibility for obesity and metabolic-related pathologies in adult offspring [2]. Maternal nutritional programming by cafeteria diet (CAF) in murine models sets metabolic alterations including impaired insulin sensitivity, hypertension, endothelial dysfunction, increased adiposity [3,4,5], and altered appetite regulation (hyperphagia) [2,6].

Food intake is actively regulated by ghrelin, which is the only known appetite-inducing peptide produced by endocrine cells of the gastric mucosa [7,8]. Ghrelin induces a powerful orexigenic signal by activating the secretagogue receptors of growth hormone (GHSR) expressed in the hypothalamic POMC of the arcuate nucleus (ARC) and the NPY/AgRP neurons of the paraventricular nucleus (PVH) [7,8]. Maternal programming by overnutrition increases plasma ghrelin levels in both dams and their offspring [9]; Additionally, neonatal overfeeding disrupts ghrelin signaling [10] and induces overweight into adulthood in both males and females [11,12]. Maternal nutritional programming by overnutrition also stimulates proliferation of neuroepithelial and neuronal precursor cells in the hypothalamus during the embryonic period, leading to differentiation and proliferation of orexigenic peptide-producing neurons [13]. Furthermore, a recent clinical study demonstrated that the maternal prenatal lipid profile was associated with the offspring’s eating behavior and energy intake [14]. This evidence proposes that maternal nutritional programming might set an orexigenic phenotype in the offspring by increasing plasma ghrelin levels and orexigenic neuronal expression in the hypothalamic nucleus.

Maternal obesity or maternal overnutrition is also associated with a positive inflammatory profile, known as metabolic inflammation. For instance, free fatty acids accumulation in plasma during maternal programming promote a central and peripheral inflammatory response through toll-like receptor 4 (TLR-4) activation [15,16,17]. In addition, clinical evidence showed substantial increased expression of TLR-4, IL-6, and IL-8 in the placenta of obese women [18], and TLR-4 ablation in the ARC nucleus of murine models prevents obesity [19]. 

In the brain, hypothalamic microglia actively responds to accumulation of saturated fatty acids following caloric exposure in murine models [11,12]. We have reported that the saturated lipid palmitic acid leads to the activation of the TLR-4-TBK1 pathway in the hypothalamus of obese murine models, which correlates with insulin resistance [20]. Of note, initial reports identified that chronic microglia activation following caloric exposure correlates with ghrelin resistance in the hypothalamus [21]. Conversely, genetic ablation of microglia leads to anorexia and weight loss [22]. Of importance, seminal studies have identified that plasma lipid profile selectively regulates ghrelin sensitivity. For instance, lipid infusions in humans suppresses the GHSR effects of ghrelin [23]. Additionally, in vitro studies identified that prolonged exposure to unsaturated fatty acids activates ghrelin sensitivity, potentially due to an increase in the GHSR in lipid rafts [24]. This evidence suggests that unsaturated or saturated plasma lipid profiles promote microglia activation, leading to positive or negative ghrelin sensitivity, respectively. It is unknown whether overnutrition during maternal programming primes hypothalamic ghrelin signaling in offspring, promoting increased food intake in adulthood. The current study was designed to identify the effect of maternal nutritional programming by caloric exposure on ghrelin-induced activation of hypothalamic neurons and food intake regulation in the offspring.

## 2. Materials and Methods 

### 2.1. Reagents and Antibodies

Reagents and antibodies used in our experimental design are showed in Table 1 and Table 2, respectively. 

### 2.2. Animals and Housing

All the experiments were performed using two-month-old wild-type female Wistar rats (initial body weight 200–250 g). Animals were handled according to the NIH guide for the care and use of laboratory animals (NIH Publications No. 80–23, revised in 1996). We followed the Basel Declaration to implement the ethical principles of Replacement, Reduction and Refinement of experimental animal models. Our study was approved by the local Animal Care Committee (BI0002). Rats were housed individually in Plexiglas-style cages, maintained at 20–23 °C in a temperature-controlled room with a 12-h light/dark cycle. Water was available ad libitum in the home cage. Food availability is described below.

### 2.3. Diets

The standard chow diet formula contained 57% carbohydrates, 13% lipids, and 30% proteins, caloric density = 3.35 kcal/g (LabDiet, St. Louis, MO 63144, 5001, Cat. D12450B). Cafeteria (CAF) diet was made of liquid chocolate, biscuits, bacon, fries potatoes, standard diet, and pork paté based on a 1:1:1:1:1:1:2 ratio, respectively; total calories 3.72 kcal/g in 39% carbohydrates, 49% lipids, 12% proteins, and 513.53 mg of sodium, caloric density = 3.72 kcal/g, as we reported before [5]. It is important to note that the CAF diet simulates the feeding habits of human populations in North America [25].

### 2.4. Maternal Nutritional Programming Model

**Figure 1:**
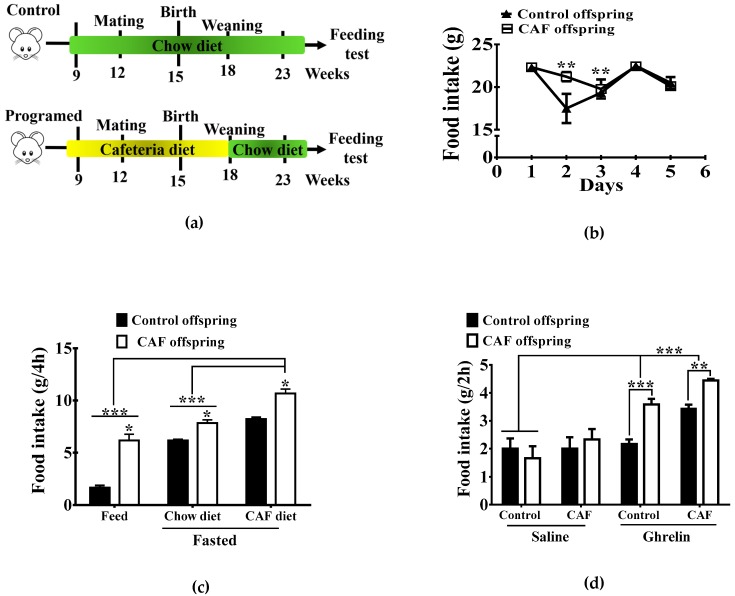
Effect of maternal nutritional programming on food intake in male offspring. (**a**) Maternal programing was performed by exposing Chow or CAF diet for nine weeks including pre-pregnancy, pregnancy and lactation. After weaning the offspring of both (CAF and Chow diets) was exposed to Chow diet for 5 weeks, by two months of age (week 23) we performed the feeding test. (**b**) Daily food intake by both Chow offspring and CAF diet offspring. (**c**) Chow and CAF diet consumption during 4 h in offspring after fasting for 16 h and refeeding. (**d**) Food intake for 2 h after administration with ghrelin 0.2 μg/Kg SC. (control diet group *n* = 10–12; cafeteria diet (CAF) group *n* = 10–12; the graphs show normalized data of the mean ± S.E.M. Two-way ANOVA followed by Tukey multiple comparation test; * *p* <0.05, ** *p* <0.01, *** *p* <0.001).

Programing and mating experiments were performed using 12-week-old male and 10-weel-old virgin female Wistar rats. Animals were acclimated to the animal facility seven days prior to the nutritional programming protocol in standard conditions with ad libitum access to food and water. Female rats (*n* = 6) were randomized into two batches of three animals each, one for the control chow diet and the second for the CAF diet, as we reported [5]. After randomization, female rats were exposed ad libitum to specific formula diets three weeks before mating. Rats were mated with age-matched Wistar males for two days and males were removed from the home cage. Pregnancy diagnosis was performed in females after mating by vaginal plug. Female rats lacking copulation plugs were returned to the home cage for a second mating. Pregnant rats were kept on the same diet until birth and lactation. Male offspring from mothers exposed to Chow or CAF diets were weaned at post-natal day 21, grouped into 10–12 subjects per group and exposed to control Chow diet (Control Chow and CAF programmed groups) for nine weeks. During the experiment, body weight and food intake were measured weekly (Figure 1a).

### 2.5. Analysis of Ghrelin Signaling for Chow and CAF Exposure in Offspring

The offspring from mothers exposed to Chow (*n* = 10–12) or CAF (*n* = 10–12) diet were fasted for 16 h by removing their food at 18:00 PM. To measure total food intake, Chow and CAF diets were weighed and placed inside the cages, where they were left for 4 h, after which food was removed and weighed. Additionally, after removing the food, Chow or CAF programmed offspring were injected intradermically with 0.2 micrograms/kg of ghrelin (*n* = 10–12) or saline (*n* = 10–12), and food was placed in their cages for 2 h (see Table 1 for reagents). Rats were allowed to eat ad libitum, and then food was removed and weighed.

This procedure allowed each subject to be its own control for the ghrelin effect. Next, rats were intracardially perfused and processed for immunohistochemistry against c-Fos for neuronal and Iba-1 for microglia activation (see Table 2 for antibodies), as described below.

### 2.6. Intracardiac Perfusion

After 2 h of ghrelin administration, the offspring was anesthetized by 1 mL pentobarbital (PiSA Agropecuaria) i.p. overdose. A dermal dissection was performed from the abdominal region to the upper part of the thoracic cage, exposing the heart. Then, the left ventricle of the heart was perforated following its apex and a cut was made in the right atrium in order to open the circulatory system. Rats were intracardially perfused with 250 mL 0.1 M phosphate-buffered saline (PBS) + 10 U/mL heparin followed by 250 mL paraformaldehyde 4% in PBS 0.1M (PFA) using an infusion pump (Fisher Scientific GP1000) at a flow rate of 15 mL/min. The brains were collected, and samples were stored in 4% PFA + PBS 0.1 M at 4 °C for 24 hours and changed to 10%, 20%, and 30% sucrose before cutting. We obtained 40-μm coronal sections in the rostral-caudal direction between bregma −1.58 and −1.94 mm for the ARC, and between bregma −0.70 and −0.94 mm for the PVN using a cryostat; the sections were processed for immunohistochemistry as described below. Anatomical limits of each brain region were identified using the Paxinos and Watson Atlas [26]. 

### 2.7. Tissue Sample Collection

The second batch of offspring (*n* = 10–12 each) were sacrificed by decapitation at nine weeks of age. Blood samples were collected in 500-μL tubes (Beckton Dickinson) and plasma fraction was isolated by centrifugation at 4 °C and frozen at −80 °C. ARC and PVN-DMN of hypothalamus were dissected and frozen immediately at 80 °C for Western blot analysis.

### 2.8. Stereotaxic Surgery and I.C.V. Ghrelin Administration

A third batch of rats was selected for stereotaxic brain surgeries to selectively stimulate the hypothalamic region. In brief, all surgeries were carried out using aseptic techniques and animals were injected with ketamine (100 mg/kg, i.p.) + xylazine (10 mg/kg, i.p.) to induce anesthesia and analgesia, respectively. By eight weeks of age, male rats (*n* = 36) were implanted with a cannula in the third ventricle following the stereotaxic coordinates, AP: −2.56, mL: 0, DV: −9.4 according to the rat brain atlas [26]. The animals were allowed to recover for seven days and placed into three experimental groups (*n* = 8/group) for five days i.c.v administration of: 1) artificial cerebrospinal fluid (ACSF) (Control), 2) 40 μg/μL palmitic acid (PAL), and 3) 2 μg/μL lipopolysaccharide (LPS) (*Escherichia coli* 0111: B4). We performed a single i.c.v administration of ACSF, PAL or LPS using an infusion pump at 2 μL flux rate. We quantified total food intake per day as well as glucose and insulin following the i.c.v administration of 1μg/μL ghrelin. 

### 2.9. Glucose and Insulin Tolerance Test (GTT, ITT) and Ghrelin Sensitivity Assessments

Following i.c.v administration, males were fasted for 8 or 12 h and were intraperitoneally injected with 1 U of insulin/100 g or 40% glucose/kg body weight, respectively. Blood glucose levels were quantified at 0, 15, 30, 45, 60, 90, and 120 min, as described previously [5]. A week after GTT and ITT assessments, rats were i.c.v. injected with ghrelin (1 μg/μL) and we quantified total food intake for two hours. Subjects were transcardially perfused as described and processed for immunohistochemistry analysis as described below. 

### 2.10. Microglia Primary Culture and Treatments

Cerebral cortices from the postnatal day 2 (P2) Wistar rat pups were surgically dissected, harvested, the meninges and blood vessels were removed, and the parenchyma minced and triturated in Leibovitz’s L-15 Medium (Thermo Fisher Scientific, Waltham, Massachusetts) + 0.1% bovine serum albumin (BSA) with 4.5 g/L glucose, 100 U/mL penicillin, and 0.1 mg/mL streptomycin. Suspended cells were filtered (70 mm) and plated on T-75 Flasks containing 10 mL of Dulbecco’s Modified Eagle’s medium (DMEM) (SIGMA, San Luis, MO, USA); media were replenished every two days, resulting in mixed glial monolayers. Two weeks later, the flasks were shaken (200 rpm) for two hours (37 °C) to specifically release microglia.

Microglia culture were 70–80% confluent and stimulated for 24h with one of each fatty acids as follow: 100 µM palmitic acid, 100 µM palmitoleic acid, 100 µM stearic acid and 100 µM linoleic acid (Sigma-Aldrich, San Luis, MO, USA), 25 μM N-hexanoyl-D-sphingosine (C6) or 0.1 μg/mL LPS. Culture medium was recovered to perform the IL-6, IL-1β, and TNF-alpha ELISA tests. All fatty acids were first solubilized in DMEM media containing 10% of free fatty acid BSA, then administered in each well. Microglia were harvested and total protein was extracted using lysis buffer for Western blot analysis as described below.

### 2.11. Cytokine Measurements

Levels of IL-6, IL-1β, and TNF- alpha in culture medium of microglia following fatty acids or LPS stimulation were measured by ELISA (Sigma-Aldrich, St. Louis, MO, USA), following the manufacturer’s instructions.

### 2.12. Immunohistochemistry Analysis by Confocal Imaging

Frozen brain sections were air dried at room temperature (RT) for one hour to prevent sections from falling off the slides during antibody incubations. Afterwards, the slices were washed three times for 5 min with 1X PBS + 0.1% TritonX-100 (PBST) and the slices were blocked in PBST + 10% goat serum for an hour. Subsequently, brain sections were incubated with PBST + 5% goat serum with the primary antibody anti-Iba-1 (1:200) or anti-c-Fos (1:1000) at 4 °C overnight. Subsequently, the slices were washed in PBST three times and then incubated for three hours with the secondary antibodies Alexa Fluor 488 Rabbit Goat Anti-Rabbit (IgG) for C-Fos or Alexa Fluor 546 Goat Anti-Rabbit (IgG) for Iba-1, each diluted in PBST + 5% goat serum (1:1000). Afterwards, brain sections were washed three times with PBST and air-dried at RT. Finally, brain sections were mounted using Vectashield with DAPI (Vector Laboratories, Burlingame, CA, USA) on coverslips.

### 2.13. Western Blot Analysis

Microglia culture samples and ARC and PVN-DMN hypothalamic biopsies were incubated in lysis buffer solution (150 mM NaCl, 25 mM Tris–HCl pH 7.5, containing 50 mM NaF, 10 mM NaP2O7, 1 mM Sodium orthovanadate, complete protease inhibitor cocktail (Roche, Mannheim, Germany) 0.5% Triton X-100) followed by sonication (for 5 s at 1500 Hz on ice). Samples were centrifuged for 10 min at 1500× *g* and protein concentration were determined by the bicinchoninic acid (BCA) assay at a concentration of 40 μg/μL. Samples were mixed with Laemmli buffer and then heated t 90 °C for 5 min and subjected to SDS–PAGE. Proteins were electrophoretically transferred to nitrocellulose membranes. The membrane was then blocked for 2 h at RT in TBS-T buffer (10mM Tris, 0.9% NaCl, 0.1% Tween 20, pH 7.5) containing 5% BSA. Membranes were incubated overnight with primary antibodies at 4 °C: TBK1 (1:1000), TBK1-pSer172, anti-NF-κB (1:1000), NF-κB-pSer536 (1:1000), anti-actin (1:2000). Membranes were washed (4 times/5 min) in TBS-T and incubated for 1 h with horseradish peroxidase (HRP)-conjugated secondary antibody. Proteins were detected by ECL, which were read employing the ChemiDoc™ XRS+ System (BIO-RAD) and quantified densitometrically with the 1.31V ImageJ software (Wayne Rasband, National Institutes of Health, Bethesda, MD, USA). 

### 2.14. Statistical Analysis

Statistical analyses were conducted using the Prism 7 GraphPad software. Western blot data were analyzed using the unpaired Student’s *t*-test. We used two-way ANOVA followed by Tukey for multiple comparations. Data are presented as mean ± SD. The significance levels displayed on figures are as follows: * *p* < 0.05, ** *p* < 0.01, *** *p* < 0.001.

## 3. Results

### 3.1. Maternal Nutritional Programming Exacerbates Ghrelin Sensitivity in Offspring, Leading to an Increase in Food Intake

First, we aimed to identify the effect of maternal nutritional programming by caloric exposure on basal and ghrelin-induced food intake regulation in the offspring. We found that offspring showed a significant transient increase of food intake by day two during the five days schedule, with no changes on total basal food intake (Figure 1b). Additionally, offspring programmed by the CAF diet exhibited an increase in Chow and CAF food intake after 14 h fasting when compared with offspring exposed to Chow diet during programming (Figure 1c). Of note, incentive food intake behavior in the programmed offspring was sensitive to ghrelin administration. We found that subcutaneous ghrelin injection significantly increased Chow and CAF diet intake in offspring programmed by maternal CAF compared with Chow programmed offspring (Figure 1d). These results suggest that maternal programming by CAF diet actively promotes food intake by potentially priming ghrelin sensitivity in offspring. 

### 3.2. Maternal Programming by CAF Exposure Sets Activation of Hypothalamic Ghrelin-Sensitive C-Fos Neurons in Offspring 

**Figure 2:**
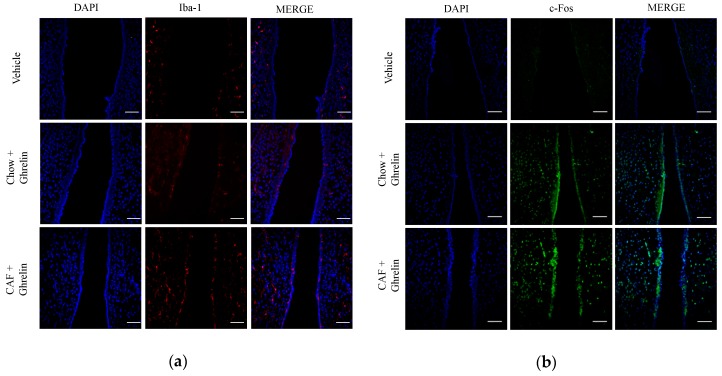
Maternal programming leads to microglia activation and c-Fos response in hypothalamus. Offspring was programmed by CAF diet exposure as previously described, and 0.2 micrograms/kg ghrelin was intradermically administered, and subjects were intracardially perfused with 0.1M PBS + heparin followed by PBS + 4% PFA. Hypothalamic sections were obtained using a cryostat, according to the Paxinos and Watson Atlas. Immunofluorescence to identify microglia activation was performed using the Iba-1 antibody (1:200) following by the secondary antibody Alexa Fluor 546 Goat Anti-Rabbit (IgG) (1:1000) (**a**). c-Fos activation was identified by anti c-Fos (1:1000) and Alexa Fluor 488 Rabbit Goat Anti-Rabbit (1:1000) (**b**). Brain sections were mounted using Vectashield with DAPI (Vector Laboratories) on coverslips. (*n* = 3). PBS, phosphate-buffered saline, PFA, paraformaldehyde.

Next, we sought to identify if ghrelin sensitivity correlated with neuronal response and microglia activation in the hypothalamic ARC nucleus. We used a 120-min time-frame schedule following systemic ghrelin administration to identify central neuronal c-fos activation in the ARC nucleus leading to food intake response, as reported [27]. We found that offspring programmed by a maternal CAF diet exposure showed substantial microglia activation evidenced by an increase in the Iba-1 marker (Figure 2a). Additionally, CAF diet exposure promoted a higher neuronal c-fos expression in ARC when compared with matched controls (Figure 2b). Together, these results confirm that maternal programming is able to sensitize central neuronal activation in the hypothalamus of offspring following systemic ghrelin administration, which correlates with central microglia activation. 

### 3.3. Saturated Lipids Activate a Pro-Inflammatory Stage in Microglia

**Figure 3:**
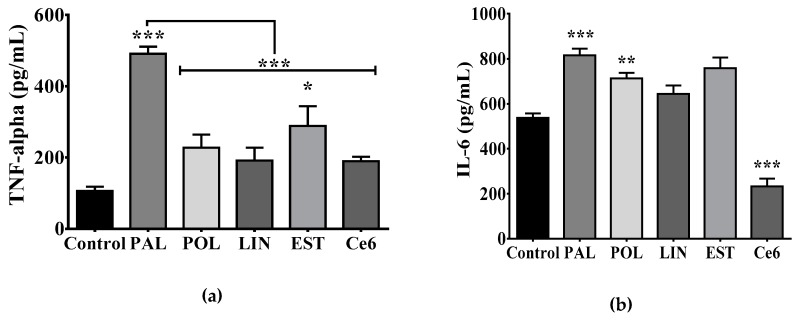

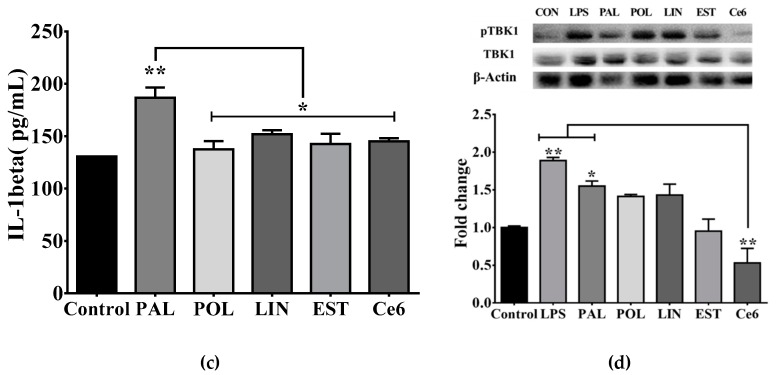
Palmitic acid incubation leads to cytokine production and TBK1 pathway activation in primary microglia. (**a**) TNF-alpha secretion, (**b**) IL-6 secretion or (**c**) IL-1β secretion by primary microglia culture following 1% BSA-FFA (Control); 100 μM palmitic acid, palmitoleic acid, linoleic acid or stearic acid or 25 μM C6 ceramide incubation for 24h. TNF-alpha, IL-6 and IL-1β secretion were quantified by ELISA following the manufacturer’s instructions (*n* = 4). (**d**) TBK1 phosphorylation following saturated and unsaturated fatty acids stimulation was identified using western blot analysis. The graphs show normalized data of the mean ± S.E.M. Two-way ANOVA followed by Tukey multiple comparation test; * *p* < 0.05, ** *p* < 0.01, *** *p* < 0.001). TBK1, TANK-binding kinase 1, BSA-FFA, Bovine serum albumin-free fatty acids (FFA).

To identify whether positive energy balance in offspring after programming selectively displayed microglia activation and ghrelin sensitivity, we initially characterized potential lipid species showing a pro-inflammatory profile using a microglia in vitro system. We performed saturated or unsaturated fatty acids stimulation for 24 h and quantified TNF-alpha, IL-6, and IL-1β release, as described. We found that the C16:0-saturated palmitic acid incubation efficiently promotes TNF-alpha, IL-6 and IL-1β release when compared with control, whereas the C18:0 long-chain stearic acid promotes TNF-alpha release with no changes in IL-6 and IL-1β (Figure 3a–c). Additionally, palmitoleic acid (C16:1), the monounsaturated form of palmitic acid promoted the production of IL-6, whereas the C6 ceramide (C18:1/6:0) decreased it (Figure 3b). Finally, the polyunsaturated linoleic acid (C18:2) incubation did not show changes in the pro-inflammatory profile of microglia (Figure 3a–c). 

We previously reported that palmitic acid leads to the TLR4-TBK1 pathway activation in the hypothalamus of obese murine models, which correlates with systemic insulin resistance [20]. We identified whether stimulation with saturated and unsaturated fatty acids lead to TBK1 activation in microglia cultures. Our results show that both the positive inflammatory reagent, LPS, and palmitic acid elicit a significant activation of the TBK1 pathway in microglia cells (Figure 3d). Of importance, the C6 ceramide blocks the TBK1 pathway activation (Figure 3d), which correlates with no changes in TNF-alpha and IL-1β release (Figure 3a,b) with a decrease in the IL-6 release (Figure 3c). Our results confirmed the saturated palmitic acid as a reliable lipid species leading to pro-inflammatory state in microglia cells.

### 3.4. Hypothalamic Palmitic Acid Inoculation Promotes Increase in Total Food Intake

**Figure 4:**
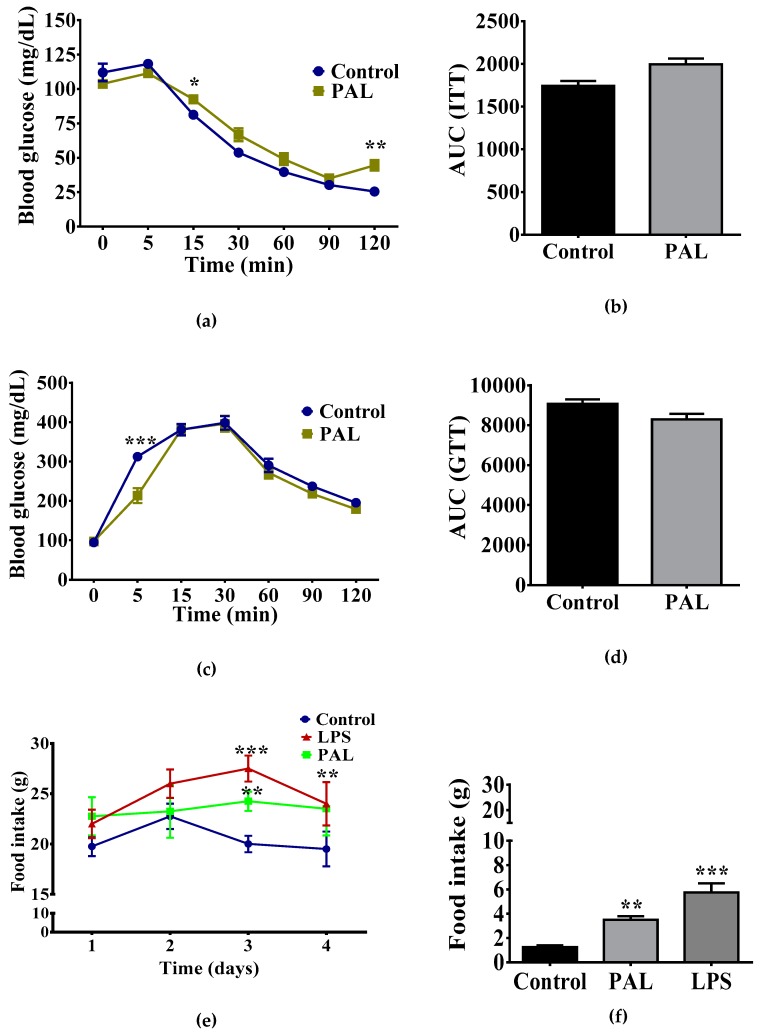
Chronic i.c.v. palmitic acid administration sensitizes ghrelin signaling leading to food intake increase. ITT and area under the curve (AUC) (**a**–**b**) and GTT and AUC (**c**–**d**) were analyzed following i.c.v of 2 μg/mL LPS or 40 μg/μL palmitic acid administration for five days. Daily food intake quantification following palmitic acid, LPS or ACSF administration (**e**). Ghrelin-sensitive food intake was analyzed by day 5 after 2 h, 1 μg/μL ghrelin i.c.v. administration (**f**). The graphs show normalized data of the mean ± S.E.M., Student’s *t*-test, * *p* < 0.05). (*n* = 4, the results are shown as the mean ± S.E.M. Two-way ANOVA followed by Tukey multiple comparation test; * *p* < 0.05, ** *p* < 0.01, *** *p* < 0.001). LPS, Lipopolysaccharides, ACSF, artificial cerebrospinal fluid.

We determined whether central administration of the pro-inflammatory modulator palmitic acid disrupted plasma glucose homeostasis and/or food intake response. We found that i.c.v administration of palmitic acid for 5 days partially increased plasma glucose levels following insulin administration, however, it did not change total glucose homeostasis evidenced by area under the curve (AUC) quantification (Figure 4a,b). Additionally, we found no changes in glucose homeostasis addressing by the GTT (Figure 4c,d). 

Next, we hypothesized that the pro-inflammatory modulator palmitic acid might reproduce the increase in food intake found in the offspring of mothers exposed to CAF diet. We administered LPS, palmitic acid or vehicle via i.c.v for five days and we quantified basal food intake and food intake following i.c.v administration of ghrelin (1 μg/μL). We found a time-dependent increase of daily total food intake during LPS or palmitic acid schedule when compared with ACSF administration (Figure 4e). Additionally, we identified that five days of palmitic acid administration results in a 3.5-fold increase of food intake following i.c.v. administration of ghrelin when compared with the control group (Figure 4f). Of note, i.c.v. administration of the pro-inflammatory reagent LPS reproduces the total food intake induced by ghrelin administration (Figure 4f). These results support the hypothesis that saturated lipids sensitize hypothalamic ghrelin signaling, leading to exacerbation of food intake.

### 3.5. Hypothalamic Saturated Lipids Stimulation Activates Microglia and Ghrelin-Sensitive Neurons 

**Figure 5:**
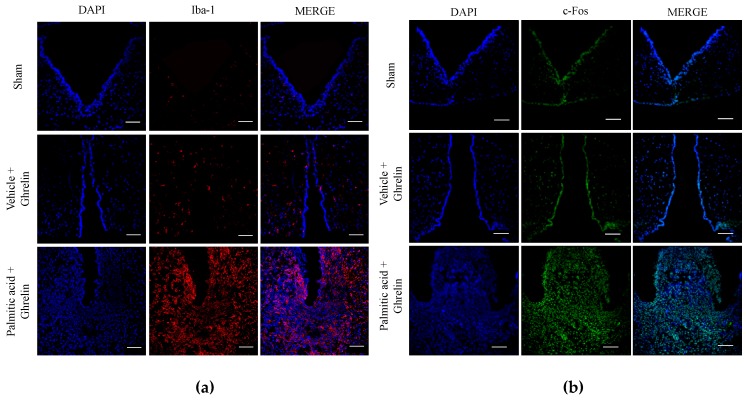

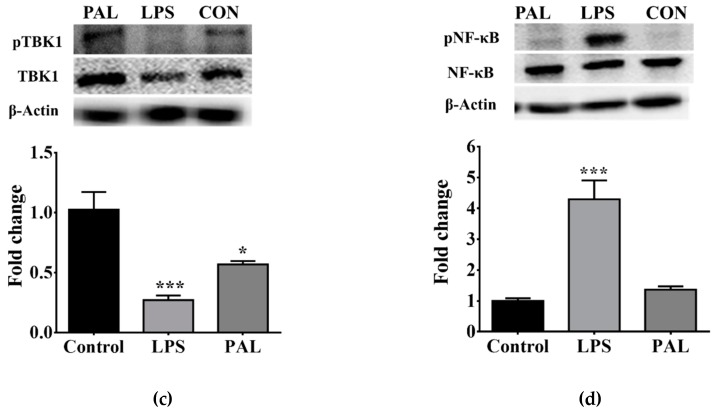
Palmitic acid promotes microglia activation and c-fos response in hypothalamus. Rats were i.c.v. administered with, ACSF, 2 μg/μL LPS or 40μg/μL palmitic acid for 5 days and vehicle or ghrelin was injected into the third ventricle by day 5. Immunofluorescence against Iba-1 marker (microglia activation) (**a**) or c-fos activation were performed as described in Figure 4 (**b**). (**c** and **d**) Changes in TBK1 and NF-κB phosphorylation in the ARC of hypothalamus were identified using western blot analysis following i.c.v. ACSF, LPS or palmitic acid administration for five days (*n* = 4 per group). The graphs show normalized data of the mean ± S.E.M. Two-way ANOVA followed by Tukey multiple comparation test; * *p* < 0.05, *** *p* < 0.001). TBK1, TANK-binding kinase 1, BSA-FFA, Bovine serum albumin-free fatty acids, ACSF, cerebrospinal fluid, PAL, palmitic acid, LPS, lipopolysaccharide.

At this final stage, we integrated the in vivo and in vitro results identified in Figure 1, Figure 2, Figure 3 and Figure 4, supporting the effect of saturated palmitic acid on microglia activation and increased food intake in programmed offspring. We determined that palmitic acid promoted hypothalamic microglia activation and that it correlated with ghrelin-sensitive neuronal activation. We found that i.c.v. palmitic acid administration for five days following by ghrelin stimulation promoted a significant increase of microglia activation in the ARC of the hypothalamus, evidenced by immunofluorescence stain of the IBA1 marker when compared with the vehicle (Figure 5a). We also confirmed that palmitic acid promoted an increase in the c-fos immunosignal, confirming neuronal activation in the ARC nucleus (Figure 5b).

Finally, microglia activation in the ARC nucleus of the hypothalamus after palmitic acid administration was also evaluated by analyzing the TLR4-TBK1 pathway. Western blot analysis showed a decrease of the nuclear factor kappa B (NF-κB) phosphorylation following palmitic acid stimulation, which is also promoted by LPS (Figure 5c). As expected, LPS administration promoted a four-fold increase of NF-κB phosphorylation in the ARC of the hypothalamus and no changes were found after stimulation with palmitic acid (Figure 5d).

## 4. Discussion

Extensive evidence has confirmed that maternal obesity or maternal overnutrition during pregnancy and lactation negatively modulate peripheral and central pathways disturbing basal energy homeostasis and appetite regulation in the offspring [1,2]. We, and others, have identified that maternal nutritional programming by CAF diet exposure in murine models sets metabolic alterations [3,4,5] and defective behaviors, including addiction-like behavior [6,28], which potentially contributes to hyperphagia [2]. Here, by using in vitro and in vivo experimental approaches we suitably identified the lipotoxic effect of the saturated lipid species palmitic acid as a potential trigger to promote hypothalamic microglia activation and actively sensitize the central effects of ghrelin on food intake. Notably, we also discovered that maternal programming by CAF diet exposure also reproduces the effect of palmitic acid in the hypothalamus by priming the central ghrelin response for feeding in the offspring. Based on our results, we propose that palmitic acid might be considered a lipid species capable of dynamically priming hypothalamic ghrelin sensitivity to food intake in the offspring of mothers programmed by caloric diets.

A key contribution of our study is that nutritional programming by CAF exposure increases basal feeding response and ghrelin-sensitivity in the offspring. Initially, daily food intake was not increased on CAF diet –programmed groups, however, we did observe hyperphagia following plasma increase of ghrelin levels by fasting or SC ghrelin injection. As already proposed, maternal programming by caloric exposure increases ghrelin levels in the offspring [9], which may contribute to the hyperphagic phenotype during fasting in our model. On this context, in a parallel scenario we identified that maternal programming primes hypothalamic neuronal activation in the offspring, evidenced by an increase in c-fos expression in the ARC nucleus, which is also exacerbated by i.c.v. or subcutaneous ghrelin administration. This suggests that priming of central ghrelin signaling exacerbates feeding in the context of fasting or pharmacologic plasma ghrelin increase. Our data agree with the effects of maternal nutritional programming by caloric diets in murine models, which stimulates the proliferation of orexigenic peptide--producing neurons [13]] and leads to hyperphagia in the offspring [2]. Notably, the effect of plasma lipidomic profile leading to food intake in humans suggests that the higher prenatal triglycerides plasma concentrations in humans were associated with higher food responsiveness in offspring at 5 years old [14]. These results are also in line with murine models showing that increased perinatal triglyceride concentrations correlate with hyperphagia in the offspring [29]. Together, these results propose that nutritional programing by CAF diet primes the ghrelin response for Chow and CAF feeding in the offspring. 

Maternal obesity or maternal overnutrition promotes free fatty acid accumulation in plasma, which is associated with central and peripheral inflammatory response, potentially throughout the TLR-4 pathway [13,14,15]. In fact, TLR-4, IL-6, and IL-8 expression have been identified in the placenta of obese women when compared with lean women [16]. In murine models, plasma accumulation of saturated fatty acids in diet-induced obesity activates hypothalamic microglia [18,19]. In fact, there is an interactive crosstalk integrating central and peripheral immunity regulating hypothalamic nodes for metabolic and feeding homeostasis [16]. For instance, we have reported that the saturated lipid palmitic acid leads to the TLR-4-TBK1 pathway activation in the hypothalamus of obese murine models, which correlates with insulin resistance [19]. Here we hypothesized that maternal programming by CAF exposure sets a plasma lipotoxic profile in the offspring, leading to central microglia activation. Our experimental in vitro data allowed us to propose that, in contrast to the unsaturated lipid species such as the palmitoleic acid, linoleic acid or even, the C6 ceramide, the saturated lipid species palmitic acid, is an effective pro-inflammatory mediator in microglia activating the IL-6, IL-1β and TNF-alpha cytokines release. Additionally, LPS stimulation in microglia cells closely reproduces the pro-inflammatory profile found during palmitic acid stimulation, supporting the negative effect of palmitic acid. 

One of the most outstanding results in our study is the major effect of hypothalamic palmitic acid inoculation on sensitizing ghrelin response for food intake. Ghrelin has been reported as a molecule that prevents the lipotoxic effects of palmitic acid stimulation in diverse cell types, including hepatocytes, pancreatic β-cells, and myoblasts [30,31,32]. In fact, ghrelin also exerts immunomodulatory effects on macrophages, T lymphocytes and microglia, guiding these cells towards an anti-inflammatory phenotype during obesity-induced inflammation [16,33]. In addition, ghrelin regulates by antagonizing TNF-α/NF-κB and TLR-4 signaling pathways [16,34] associated whit metabolic inflammation on the brain [35]. In any case, does hypothalamic microglia activation by palmitic acid explains the priming ghrelin response in the offspring programmed by CAF diet exposure? Programming the offspring with CAF diet exposure closely replicates microglia activation in the ARC nucleus of the hypothalamus, evidenced by an increase in the Iba-1 marker staining. Our results agree with previous data showing that systemic LPS inoculation activates central microglia [11,12,36]. In the beginning, clinical evidence, reported that the microglial inhibitor minocycline induced weight loss as a major side-effect [37]. Experimental basic evidence confirms that transient pharmacologic microglia depletion in murine models leaded to food intake decrease and weight loss [22]. Supporting our findings, neonatal overfed rats show hypothalamic microglia activation positive for Iba-1 and IL-6 and NF-κB expression, which correlates with accelerated weight gain [38]. However, additional experimental evidence does not support this scenario by showing that LPS, certain cytokines (e.g., IL-1β and TNF-α) and dietary lipids sets, in fact, a pathological state known as sickness-associated anorexia [16,39], which actively induces IL-1β and TNF-α mRNA expression in the ARC nucleus of the hypothalamus [40]. Additionally, weight loss and anorexia in mice do not show a peripheral or central pro-inflammatory phenotype [22], and pharmacologic microglia ablation does not promote changes in mice body weight [41]. 

We suggest that an explanation to this discrepancy might be associated with a time-dependent pro-inflammatory profile including LPS, palmitic acid and dietary lipids in our model, and potential hormone and metabolic profiles modulating central and peripheral immune activation. For instance, inflammation and gliosis are detected in rat and mouse ARC nucleus within the first few days following high-fat diet exposure, before obesity develops [42], suggesting that a positive inflammatory profile promotes central leptin and insulin resistance [16], which potentially leads to an increase in plasma leptin levels favoring weight gain [43]. Additionally, hypothalamic microglia activation by lipid over supply might prime ghrelin sensitivity, potentially due to an increase in the GHSR into lipid rafts [24]. In any case, based on these data we propose that during the maternal CAF diet programming, palmitic acid might be one lipid species capable of promoting central microglia activation in the offspring.

Our study still lacks evidence if maternal programming by a CAF diet leads to plasma and brain increases of palmitic acid to support its effect on microglia activation and ghrelin sensitivity. Reports have shown that mice exposed to a western-style high fat diet for 16 weeks experienced a C16:0 saturated lipid increase in plasma and brain [44], including hippocampus [45]. In fact, an increase in plasma palmitic acid has been identified in healthy overweight subjects showing upper tertiles (T3) according to L4 visceral fat area [46]. Importantly, the direct effect of palmitic acid intake on brain function and its correlation with positive inflammatory immune profile in humans, was recently identified, showing that three-week high palmitic acid diet intake promotes an increase in the basal ganglia activation which correlates with IL-6, IL-18, and IL-1β plasma accumulation [47]. Finally, experimental evidence has also confirmed that the integrity of the blood brain barrier is compromised following western diet exposure in mice [48], which tentatively suggests that selective fatty acids incorporation into the brain might be favored during blood-brain barrier disruption and contribute to palmitic acid flux to the hypothalamus, a region lacking a blood-brain barrier.

## 5. Conclusions

Our results support the hypothesis that maternal programming by CAF diet exposure primes a hypothalamic ghrelin response leading to food intake exacerbation in male offspring. Of note, caloric programing also replicates the central microglia activation and ghrelin sensitivity found during intraventricular palmitic acid administration. We propose that palmitic acid might be considered a lipid species involved in priming of hypothalamic ghrelin sensitivity to food intake in the offspring during CAF diet exposure.

## Figures and Tables

**Table 1:** List of reagents

Reagent	Catalog	Application (conc.)	Manufacturer
**Ghrelin** Dulbecco´s Modified Eagle’s Medium high glucose	G8903 D5648	i.c.v PC	Sigma-Aldrich, St. Louis, MO, USA Sigma-Aldrich, St. Louis, MO, USA
L-15 Medium (Leibovitz)	L4386	PC	Sigma-Aldrich, St. Louis, MO, USA
Palmitic acid	P0500	i.c.v. and PC	Sigma-Aldrich, St. Louis, MO, USA
Palmitoleic acid	P9417	PC	Sigma-Aldrich, St. Louis, MO, USA
Linoleic acid	L1376	PC	Sigma-Aldrich, St. Louis, MO, USA
Stearic acid	S47S1	PC	Sigma-Aldrich, St. Louis, MO, USA
N-hexanoyl-D-esfingosin	H6524	PC	Sigma-Aldrich, St. Louis, MO, USA
lipopolysaccharide (LPS) *Escherichia coli* 0111: B4	L2630	i.c.v. and PC	Sigma-Aldrich, St. Louis, MO, USA
Rat TNF-α ELISA Ready-SET-Go!	88-7340	ELISA	eBioscience, San Diego, CA, USA
Rat IL-6 ELISA kit	RAB0311	ELISA	Sigma-Aldrich, St. Louis, MO, USA
Rat IL-1β ELISA kit	RAB0277	ELISA	Sigma-Aldrich, St. Louis, MO, USA

PC: primary microglia cell culture; i.c.v: intracerebroventricular injection.

**Table 2:** List of antibodies

Antibody	Catalog	Application (conc.)	Host	Manufacturer
Anti-NAK	ab40676	WB (1:1000)	Rabbit	abcam, Cambridge, MA, USA
Anti-p-NAK S172	ab109272	WB (1:1000)	Rabbit	abcam, Cambridge, MA, USA
Anti-p-NF-κB p65 S536	3033S	WB (1:1000)	Rabbit	Cell Signaling, Beverly, MA, USA
Anti-p- NF-κB p65	8242S	WB (1:1000)	Rabbit	Cell Signaling, Beverly, MA, USA
Anti- β-Actin	8457P	WB (1:5000)	Rabbit	Cell Signaling, Beverly, MA, USA
Anti-rabbit IgG-HRP	sc-2370	WB (1:1000)	Cow	Santa Cruz Biotech., Dallas, TX, USA
Alexa fluor 488 anti-rabbit	A-11034	IF (1:1000)	Goat	Thermo Fisher Scientific, Waltham, MA, USA
Alexa fluor 546 anti-rabbit	A-11035	IF (1:1000)	Goat	Thermo Fisher Scientific, Waltham, MA, USA
Anti-c-fos	ab190289	IF (1:1000)	Rabbit	abcam, Cambridge, MA, USA
Anti-Iba-1	ab178847	IF (1:200)	Rabbit	abcam, Cambridge, MA, USA

WB: western blot; IF: Immunofluorescence.
